# Hormone Replacement Therapy: Lebanese Women's Awareness, Perception, and Acceptance

**DOI:** 10.1155/2020/5240932

**Published:** 2020-06-16

**Authors:** A. Anastasia Salame, Mohammad J. Jaffal, Fatin Khalifeh, Dalia Khalife, Ghina Ghazeeri

**Affiliations:** Department of Obstetrics and Gynecology, American University of Beirut Medical Center, P.O. Box 113-6044, Beirut, Lebanon

## Abstract

**Objectives:**

Hormone replacement therapy (HRT) had been the gold standard for the treatment of menopausal symptoms until the publication of the World Health Initiative (WHI) study. After the WHI study, the use of HRT changed among the physicians and patients all over the world despite newer more reassuring data. This study aimed to investigate the knowledge and attitudes of women towards HRT and the factors affecting it for better counseling. *Study design*. A clinic-based cross-sectional study using a survey was offered to women aged 40 years and above coming to the women's health center at the American University of Beirut Medical Center (AUBMC) from October 1^st^, 2017, till March 31^st^, 2018. The questionnaire included questions about demographics and menopausal symptoms in addition to knowledge and attitudes towards menopause and HRT. *Main outcome measures*. Our main hypothesis was that women would be aware of HRT as a treatment modality; however, the majority would have a negative attitude towards its usage.

**Results:**

The response rate was 87.8%. Seventy-three percent of the respondents had already heard about HRT with 57.9% supporting the use of HRT; however, 47.9% did not know when to use it. The significant predictor for having heard about HRT and a positive attitude towards HRT were having HRT prescribed as a part of treatment and employment status, respectively.

**Conclusions:**

Lebanese women are aware of HRT as a treatment option; however, a lack of both proper information and positive attitude towards HRT use was noted.

## 1. Introduction

Menopause results from the natural variation in the hormonal profile that eventually results in the cessation of the ovarian endocrine function referred to as the climacteric change [1, 2]. Women might experience mood disturbances, depression, hot flashes, vasomotor symptoms, recurrent urinary tract infections, vaginal atrophy with alteration of the sexual life, and libido, as well as osteoporosis and cardiovascular disease [3].

Hormone replacement therapy (HRT), in the form of estrogen alone or combined estrogen and progesterone formulations, has been widely used to treat menopausal symptoms to improve women's quality of life, control their climacteric symptoms, and prevent osteoporosis [4]. However, the publication of Women's Health Initiative (WHI) controlled trial results in 2002 changed the medical and public point of view towards HRT. The WHI study assessed the benefits and risks of HRT in advanced age menopausal women and came to conclude that even though HRT decreased fracture rates and vasomotor symptoms in postmenopausal patients, it did not increase their quality of life. Moreover, it showed unexpectedly that HRT use leads to an increased incidence of cardiac events, stroke, deep venous thrombosis, and breast cancer which led to the premature closure of the study [5]. In the direct post-WHI era and being affected by the WHI results as well as by the American College of Obstetrics and Gynecology (ACOG) [6] and the U.S. Preventive Services Task Force recommendations, health care providers and obstetricians started to avoid using HRT offering menopausal patients less effective alternative treatments [7]. However, over the past years, additional studies and analyses were performed on the previously published data, pinpointing several pitfalls in the WHI study. It was concluded that the formulations of the HRT used, the time since the onset of menopause and the age at the initiation of treatment appeared to be important factors in affecting the clinical outcome. Hence, the current recommendations by the North American Menopause Society issued in 2017 specified that the clearest benefit of hormone therapy is for the treatment of vasomotor symptoms and prevention of bone loss for those at elevated risk aged younger than 60 years or within 10 years of menopause onset, if there are no contraindications [8, 9].

With the changing data concerning HRT use and its safety, research has become abundant on the knowledge and attitudes of women regarding HRT. Recently a study was published by Hamid et al. in the United Arab Emirates showing that most women had a poor knowledge regarding menopause, 35% did not use treatment for symptomatic relief, and only 27% had a good knowledge about HRT [10]. Chinese women, on the other hand, who had good knowledge about menopause but poor knowledge about HRT think that menopausal symptoms should not be treated [11]. Other reports mentioned that 40 to 83% of patients have ceased using HRT after the WHI study in other studied populations [12].

Such surveys are absent amongst the perimenopausal and menopausal Lebanese population. We aim through this survey to assess the readiness of Lebanese women to use HRT in managing menopausal symptoms when needed, irrespective of their current menopausal status. We also target to highlight the factors affecting this willingness and thus improve the counseling and the clinical outcome of the patients. An additional objective of this survey is to define the extent of impact of the WHI study on people's attitudes towards HRT.

## 2. Methods

A clinic-based cross-sectional study using a survey was offered to women coming to the women's health center at the American University of Beirut Medical Center (AUBMC) from October 1^st^, 2017, till March 31^st^, 2018. Women aged 40 years and above were approached by the research team, and after explaining the aim of our study and taking the verbal consent, they were offered the questionnaire to fill without any identifiers. The questionnaire available in the Arabic language was previously tested on 15 participants to ensure that it was legible and comprehensive.

The sample size was determined based on comparing 2 independent proportions: women above the age of 40 years old and the percentage of menopausal women experiencing menopausal symptoms. The percentage of Lebanese women aged above 40 years old according to the 2015 population statistics was estimated to be around 15.3 % of the female population (World Population Pyramid, 2015). As for the menopausal experience, around 50 % of menopausal Lebanese women will experience at least one menopausal symptom (Obermeyer, Ghorayeb, and Reynolds 1999). According to these 2 proportions, the calculated sample size would be 56. If we estimate a response rate of 40%, the number of patients to be approached is 140 with a power of 80% and a significance level of *p* < 0.05.

The questionnaire contained questions concerning different aspects of the patient's sociodemographic characteristics, parity, previous history of contraception use, menopausal status and its duration, and the menopausal symptoms. The second part of the questionnaire addressed their knowledge of HRT, including previous use, the indication for the initiation of HRT treatment and its duration, reasons behind the refusal or the cessation of the treatment, and whether they received proper counseling concerning the HRT. The last section addressed their knowledge of the WHI study results and whether it affected their decision-making when receiving HRT.

Data were described as number and percent for categorical variables, whereas the mean and standard deviation (±SD) was calculated for continuous ones. Association with knowledge and attitude of HRT was assessed by the Pearson chi-square test for categorical variables, whereas Student's *t*-test was used for continuous ones. To identify predictors of knowledge and attitude of HRT, multivariate logistic regression was performed considering the variables that were found to be statistically significant at the bivariate level, where the *p* value for entry was 0.05 and that for removal from the model was 0.10. Results are presented as odds ratios (ORs) and their corresponding 95% confidence intervals (CIs). The answers to the knowledge questions were specified on a 5-point Likert scale, ranging from “strongly disagree” to “strongly agree.” An answer was considered correct when the subject agreed with a correct statement or disagreed with an incorrect statement. Moreover, the Likert scale score was transformed to 0–100. A score of 0 indicates wrong answer, whereas a score of 100 indicates a correct answer. This scoring system was implemented on questions inquiring about knowledge and an overall score including knowledge of respondents. For all statistical analyses performed, values with a *p* value ≤0.05 were considered statistically significant. The study was reviewed and accepted by the International Review Board (IRB) at the American University of Beirut.

## 3. Results

Out of the original sample (140), 123 subjects accepted to participate in the study (response rate = 87.8%). Overall characteristics of study participants are presented in Table 1. The mean age of the respondents was 50.18 ± 8.72 years old. The majority of the participants were married (86.2%), had at least one child (85.2%), and had used oral contraceptive pills previously (78.8%).

Table 2 presents overall characteristics of HRT, as well as the percent of knowledge about HRT. Seventy-three percent of the respondents had already heard about HRT with 57.9% supporting the use of HRT as a part of treatment for women; however, 47.9% did not know when one could start using HRT. Regarding the knowledge score, the overall mean score of the respondents had correct information about HRT was 63.89 ± 13.47. The correct answers to the proposed questions are presented in Supplementary Table 1. Half of the women were prescribed HRT as a part of their treatment (51.6%); however, in only 36.7% of cases, the benefits and the side effects were discussed by the prescribing physician. Only 22.3% of the respondents were familiar with the NIH study either through TV programs or via the Internet (32.0 %, respectively). This knowledge encouraged 44% of the participants to either refuse or stop using HRT (Table 2). The variables that were significantly associated with whether the participants have heard about HRT before or not were found to menopausal status with a *p* value of 0.05 and the fact that HRT was prescribed as a treatment with a *p* value of 0.004 (Table 3). The variables that affected the attitude of the participants towards HRT significantly were the basic knowledge concerning HRT (*p* value, 0.02), namely, the belief that HRT reduces vasomotor symptoms and is good to prevent age-related symptoms with a *p* value of 0.01 and 0.03, respectively. When multivariate regression analysis was performed, it was found that the only significant predictor for having heard about HRT was having HRT prescribed as a part of treatment (OR (95% CI): 3.52 (1.49–8.31), *p*=0.004). On the other hand, employment status was found to be the significant predictor for the positive attitude towards HRT (OR (95% CI): 2.84 (1.08–7.47), *p*=0.03).

## 4. Discussion

The transition between the reproductive age and the menopausal status is one of the highlights of a woman's life. This transition is accompanied with discomfort and unusual sensations making women dread it and view it as a negative move in life. HRT were developed to ease these changes that are associated with the hormonal imbalance as well as maintenance of women's health. Data concerning the safety of HRT were contradictory, especially with the publication of the WHI study; however, recent clarifications were published by scientific bodies and guidelines were set for HRT use [5, 8, 13]. The main limiting factor for HRT use in many parts of the world nowadays remains to be the knowledge and attitudes towards HRT in the general population.

Our survey revealed that 73% are familiar with the term HRT, and almost 64% had good knowledge about the effects of HRT. This contrasts the results found in the UAE [10] and Egypt [14], 2 Arab countries where only 27% and 9% had some knowledge about HRT, respectively. Poor knowledge was also documented in a UK study where more than half of the respondents lacked the familiarity about HRT [15]. Fifty-eight percent of our respondents believed that HRT is a good treatment option when indicated, however, that a lack of knowledge of when to start the treatment was noted in 47.9% of the responders. Similar rates of positive attitudes towards HRT use was noted in the UK [15], contrary to the UAE [10] and China [11]. The lack of knowledge of when to start the treatment most likely stems from the fact that physicians are not discussing the benefits and the side effects as well as the treatment options with the patients [16, 17]. This is reflected in our survey where only 36.7% of the participants had received proper counseling about the treatment from their prescribing physicians. Earlier studies performed in Lebanon in 1999 revealed that despite 40% of Beirut-residing women seek medical advice for menopausal symptoms, only 15 % actually use HRT as a treatment [18]. Another survey performed among Lebanese gynecologists in 2005 showed that after the NIH study publication, prescriptions of HRT decreased and around 70% did counsel their patients about the breast cancer risk associated with HRT while almost 7% avoid counseling in order not to confuse their patients [19]. This finding contrasts with the low level of counseling found in our study. A possible explanation might be that the physicians are taking treatment decisions without involving the patients in the process possibly to avoid the confusion or the resilience to treatment initiation. On the other hand, a survey performed amongst German gynecologists revealed that the main obstacle towards HRT prescription is in fact patients' concerns stemming from poor media sources and disinformation [20]. As a matter of fact, only 22% of our respondents were aware of the NIH study results having Internet and TV programs as the main source of information. This emphasizes the important role that media plays affecting people's attitudes.

The fact that our respondents were acquainted with HRT was significantly related to the actual menopausal status as well as having HRT prescribed as a treatment for the bothersome symptoms. These findings emphasize the role of primary care providers in the process of diagnosis and treatment of menopausal patients. Proper counseling becomes of utmost importance in order to provide the patients with the most suitable treatment. Since our respondents did highlight the influence of media/society in general on their information, it is only logical that the physicians should properly counsel the patients about the benefits and the possible side effects of the implemented treatment, making the patient participate in the treatment plan decision. When the attitude towards HRT was studied, we found that the employment status was a significant predictor of acceptance of HRT as a treatment modality.

This might be related to a higher financial income and having insurance coverage, as shown in the 1999 study where women having insurance coverage, despite being nonsignificant, were more likely to use HRT [18].

One of the main advantages of our study is that it is the first of its type in Lebanon. Having such results will help promote proper counseling and improve treatment outcomes in patients. The patients that were targeted included the pre, peri, and postmenopausal periods, which helps understand more the different approaches to counsel. It also showed the deficient role of the primary care providers when it comes to patient management, and thus the physicians should be encouraged to participate more in the counseling process to avoid the misinformation. Of the draw backs of this study, it was performed in a tertiary care center in the capital of Lebanon. Despite the fact that this center is visited by people from all the Lebanese regions, the sample of the participants in our survey might not be representative of the whole female Lebanese population.

## 5. Conclusion

Lebanese women are aware of HRT as a treatment option; however, a lack of both proper information and positive attitude towards HRT use was noted. Menopausal status and actual HRT prescription were defined as significant predictors of HRT awareness while employment status was the only predictor for the positive attitude towards HRT.

## Figures and Tables

**Table 1:** Demographic data.

		Total *N* = 123
Age group	Mean (±SD)	50.18 ± 8.72
Number of children delivered	None	18 (14.8)
	At least one child	104 (85.2)
Marital status	Married	106 (86.2)
	Not married	17 (13.8)
Employment	Employed	56 (49.6)
	Unemployed	57 (50.4)
Education	School	32 (29.4)
	University	77 (70.6)
Smoking	Nonsmoker	89 (78.1)
	Smoker	25 (21.9)
History of OCP use	Yes	89 (78.1)
Medical history	Yes	53 (44.2)
Menstrual status	Premenopausal	53 (43.1)
	Perimenopausal	18 (14.6)
	Postmenopausal	52 (42.3)
Type of menopause in the case of menopaused female	Spontaneous	41 (78.8)
	Surgical intervention	7 (13.5)
	Medical treatment	4 (7.7)
Menopausal symptoms	None	5 (7.5)
	One symptom	10 (14.9)
	Hot flashes	4 (6.0)
	Mood swings	1 (1.5)
	Feeling more tired than usual	1 (1.5)
	Weight gain	1 (1.5)
	Vaginal dryness	1 (1.5)
	Facial hair growth	2 (3.0)
	More than 2 symptoms	52 (77.6)
Menopause duration in years	Mean (±SD)	7.99 ± 7.33

Values presented in parenthesis are in %.

**Table 2:** HRT knowledge.

		Total *N* = 123
Heard about HRT before	Yes	90 (73.2)
	No	33 (26.8)
HRT use in menopause	Positive	44 (57.9)
	Negative	32 (42.1)
When women start HRT	Before the onset of menopause	8 (6.6)
	With the onset of menopause	25 (20.7)
	One year after the onset of menopause	3 (2.5)
	When symptoms appear	10 (8.3)
	Do not know	58 (47.9)
	Never	17 (14.0)
HRT knowledge total score	Mean (±SD)	63.89 ± 13.47
HRT replaces the hormones that decrease during menopause	Mean (±SD)	64.29 ± 25.79
HRT reduces vasomotor symptoms	Mean (±SD)	70.78 ± 22.36
HRT decreases the risk of colon cancer	Mean (±SD)	54.24 ± 22.86
HRT decreases the risk of osteoporosis	Mean (±SD)	67.75 ± 26.11
HRT increases the risk of breast cancer	Mean (±SD)	65.30 ± 25.36
HRT increases the risk of uterine cancer	Mean (±SD)	61.72 ± 25.18
HRT increases the risk of heart disease	Mean (±SD)	48.53 ± 22.03
HRT increases the risk of having blood clots	Mean (±SD)	53.06 ± 22.03
HRT good solution	Mean (±SD)	69.40 ± 22.48
HRT good for preventing age	Mean (±SD)	61.72 ± 25.18
HRT prescription	Yes	63 (51.6)
Doctor discusses the benefits and side effects	Yes	18 (36.7)
Reason for using	One cause	12 (46.2)
	More than 2 causes	14 (53.8)
Symptoms improve	Yes	12 (85.7)
Source of information	Internet	9 (16.1)
	Doctor	116 (28.6)
	Society	19 (33.9)
	Side effects that you actually felt	5 (8.9)
	More than one source	7 (12.5)
Encourage treatment	Strongly agree	4 (7.0)
	Agree	21 (36.8)
	Neutral	14 (24.6)
	Disagree	14 (24.6)
	Strongly disagree	4 (7.0)
	Agree	25 (43.9)
	Disagree	32 (56.1)
Awareness of WHI source of awareness	Yes	27 (22.3)
	Internet	8 (32.0)
	Environment	3 (12.0)
	Doctor	2 (8.0)
	TV program	8 (32.0)
	More than one source	4 (16.0)
Effect on decision	Start HRT	3 (16.7)
	Refuse or stop using HRT	8 (44.4)
	Neutral	7 (38.9)

**Table 3:** Factors influencing the attitude towards HRT use.

		HRT use in menopause		
		Positive *N* = 44	Negative *N* = 32	*p* value
Age group	Mean (±SD)	50.12 ± 9.40	54.33 ± 10.18	0.14
Number of children delivered	None	4 (9.3)	4 (12.5)	0.72
	At least one child	39 (90.7)	28 (87.5)	
	None	4 (9.3)	4 (12.5)	0.03
	1–2	13 (30.2)	12 (37.5)	
	3–4	25 (58.1)	10 (31.2)	
	>4	1 (2.3)	6 (18.8)	
Marital status	Married	40 (90.9)	30 (93.8)	1.00
	Not married	4 (9.1)	2 (6.2)	
Employment	Employed	24 (60.0)	11 (39.3)	0.09
	Unemployed	16 (40.0)	17 (60.7)	
Education	School	10 (26.3)	9 (31.0)	0.67
	University	28 (73.7)	20 (69.0)	
Smoking	Nonsmoker	34 (87.2)	23 (76.7)	0.25
	Smoker	5 (12.8)	7 (23.3)	
History of OCP use	Yes	12 (30.0)	10 (31.2)	0.91
Medical history	Yes	24 (57.1)	14 (43.8)	0.25
Menstrual status	Premenopausal	19 (43.2)	12 (37.5)	0.84
	Perimenopausal	4 (9.1)	4 (12.5)	
	Postmenopausal	21 (47.7)	16 (50.0)	
Type of menopause in the case of menopaused female	Spontaneous	18 (81.8)	14 (77.8)	0.85
	Surgical intervention	2 (9.1)	3 (16.7)	
	Medical treatment	2 (9.1)	1 (5.6)	
Menopausal symptoms	None	1 (3.8)	1 (5.3)	0.13
	One symptom	7 (26.9)	1 (5.3)	
	Hot flashes	3 (11.5)	0 (0.0)	
	Mood swings	1 (3.8)	0 (0.0)	
	Feeling more tired than usual	0 (0.0)	0 (0.0)	
	Weight gain	1 (3.8)	0 (0.0)	
	Vaginal dryness	1 (3.8)	0 (0.0)	
	Facial hair growth	1 (3.8)	1 (5.3)	
	More than 2 symptoms	18 (69.2)	17 (89.5)	
Menopause duration	Mean (±SD)	8.85 ± 8.62	10.31 ± 7.29	0.63
Heard about HRT before	Yes	42 (95.5)	28 (87.5)	0.20
HRT total score	Mean (±SD)	67.02 ± 10.92	59.70 ± 15.17	0.02
HRT prescription	Yes	25 (58.1)	23 (71.9)	0.22
Source of information	Internet	2 (9.5)	4 (19.0)	0.03
	Doctor	8 (38.1)	3 (14.3)	
	Society	5 (23.8)	10 (47.6)	
	Side effects that you actually felt	0 (0.0)	3 (14.3)	
	More than one source	6 (28.6)	1 (4.8)	
Encourage treatment	Strongly agree	1 (4.5)	0 (0.0)	0.01
	Agree	12 (54.5)	3 (13.6)	
	Neutral	6 (27.3)	6 (27.3)	
	Disagree	2 (9.1)	10 (45.5)	
	Strongly disagree	1 (4.5)	3 (13.6)	
	Agree	13 (59.1)	3 (13.6)	0.002
	Disagree	9 (40.9)	19 (86.4)	
Awareness of WHI	Yes	14 (32.6)	6 (19.4)	0.21
Source of awareness	Internet	4 (30.8)	2 (33.3)	0.59
	Environment	3 (23.1)	0 (0.0)	
	Doctor	0 (0.0)	1 (16.7)	
	TV program	3 (23.1)	2 (33.3)	
	More than one source	3 (23.1)	1 (16.7)	
Effect on decision	Start HRT	2 (22.2)	0 (0.0)	0.79
	Refuse or stop using HRT	4 (44.4)	3 (60.0)	
	Neutral	3 (33.3)	2 (40.0)	

## Data Availability

Due to the sensitive nature of the questions asked in this study, the IRB team and survey respondents were assured that raw data would remain confidential and would not be shared. For further inquiries, the IRB team can be reached via the following e-mail: irb@aub.edu.lb.

## Abbreviations

HRT: Hormone replacement therapy
WHI: World Health Institute
AUBMC: American University of Beirut Medical Center.

## Supplementary Materials

Supplementary Materials Supplementary Table 1: the correct statements to the HRT-related general statements.
