# Supplementary material for: Hormone Replacement Therapy: Lebanese Women's Awareness, Perception, and Acceptance
Source: Obstet Gynecol Int. 2020 Jun 16;2020:5240932. doi: 10.1155/2020/5240932 (PMC7315264; doi:10.1155/2020/5240932)
Supplement: Supplementary Materials — Supplementary Table 1: the correct statements to the HRT-related general statements. [file 5240932.f1.docx]

| **HRT related general statement:** | **Correct answer:** |
| --- | --- |
| HRT replace the hormones that decrease during menopause  HRT reduces vasomotor symptoms  HRT decreases the risk of colon cancer  HRT decreases the risk of osteoporosis  HRT increases the risk of breast cancer  HRT increases the risk of uterine cancer  HRT increases the risk of heart disease  HRT increases the risk of having blood clots  HRT good solution  HRT good for preventing age related symptoms | True  True  True  True  True, depending on the type of the HRT used.  True, depending on the type of the HRT used.  True, when used for more than 10 years or in women older than 60 years of age.  True, depending on the type of HRT used and previous medical history.  True when indicated, taken under supervision, in women younger than 60 years and for durations less than 10 years. |

Supplementary Table 1: The correct statements to the HRT related general statements.
